# High-Yield Production of Water-Soluble MoS_2_ Quantum Dots for Fe^3+^ Detection and Cell Imaging

**DOI:** 10.3390/nano10112155

**Published:** 2020-10-29

**Authors:** Benhua Xu, Zhiqi Zhang, Peng Zhang, Li Wang, Rui Yuan, Zhenghua Ju, Weisheng Liu

**Affiliations:** 1 Chemical Engineering College, Qinghai University, Xining 810016, China; xubh1815@126.com (B.X.); faith0625823@163.com (Z.Z.); yuanruiqhdx@163.com (R.Y.); 2 Qinghai Provincial Engineering Research Center of High-Performance Light Metal Alloys and Forming, Qinghai Provincial Key Laboratory of New Light Alloys, Qinghai University, Xining 810016, China; 3 College of Chemistry and Chemical Engineering, Xi’ an Shiyou University, Xi’an 710065, China; lwang2018@xsyu.edu.cn; 4 Key Laboratory of Nonferrous Metals Chemistry and Resources Utilization of Gansu Province and State Key Laboratory of Applied Organic Chemistry, College of Chemistry and Chemical Engineering, Lanzhou University, Lanzhou 730000, China; juzhh@lzu.edu.cn

**Keywords:** water-soluble MoS_2_ quantum dots, fluorescent probe, Fe^3+^ ion sensor, living cells

## Abstract

Uniform water-soluble MoS_2_ quantum dots (WS-MSQDs) were synthesized via a sequential combination of sintering/etching/exfoliation method and solvothermal route. The obtained WS-MSQDs with average size of approximately 3.4 nm exhibited sufficient water solubility and remarkable fluorescence properties. The WS-MSQDs were utilized as a probe for detection of Fe^3+^ ions with high selectivity and specificity. Furthermore, the WS-MSQDs exhibited high fluorescence stability under different conditions. Finally, the WS-MSQDs were successfully applied for the fluorescence imaging of Fe^3+^ in living cells, which exhibited practical potential for biomedical applications.

## 1. Introduction

Two-dimensional (2D) transition-metal dichalcogenides (TMDCs) have drawn tremendous attention and shown great promise for various applications in energy storage and conversion, electronic devices and biomedicine [1,2,3]. When the lateral dimensions of 2D TMDCs are reduced into quantum dots, unique optical and electronic properties are introduced into TMDCs quantum dots owing to quantum confinement and edge effects [4]. Among the large family of TMDCs quantum dots, molybdenum disulfide (MoS_2_) quantum dots (MSQDs) are the most representative and have shown remarkable applications in biology, catalyzing, electrochemical and optoelectronic devices [5].

In widely used applications, especially biology, MSQDs need to possess good water-solubility, fine photo-stability, low cytotoxicity and excellent biocompatibility [6]. The above advantages promote their widespread applications in fluorescence sensing [7,8] and bioimaging [9,10] etc. Wu et al. have obtained MSQDs by a top-down method and the MSQDs with strong fluorescence, good cell permeability and low cytotoxicity are used as probes for in vitro imaging [11]. Recently, luminescent MS nanosheets based fluorescent sensors are applied to detect metal ions (Fe^2+^, Hg^2+^) [12]. Hence, MSQDs have potential to be novel fluorescent probes for metal ions. Among the metal ions, Fe^3+^ plays crucial roles in the growth and development of biological systems [13,14,15,16] and analysis strategies have been developed to qualitatively and quantitatively detect Fe^3+^ ion in biological systems for the early identification and diagnosis of these diseases [17,18,19]. In the past two decades, fluorescence spectrometry has gradually been drawing considerable attention to directly detecting Fe^3+^ because of its advantages of high sensitivity, excellent reproducibility, rapid response and good selectivity [20,21,22,23,24]. To date, a series of quantum dots-based fluorescent Fe^3+^ probes have been fabricated. Among them, MSQDs-based nanoprobes are representative. Ruan et al. synthesized high water-solubility MSQDs (WS-MSQDs) by the combination the ethylenediamine-assisted exfoliation and a hydrothermal process [25]. Yu et al. prepared MSQDs from a one-step hydrothermal exfoliation procedure [26]. These obtained MSQDs exhibit excellent fluorescence and are effective fluorescent probes for detecting Fe^3+^ ions with excellent sensitivity, selectivity, and fast response. However, these synthesis methods still suffer from low yield of the WS-MSQDs. Hence, it is necessary to develop a simple approach for mass production of WS-MSQDs for fully developing their properties.

In the past few years, a variety of strategies have been employed for synthesizing WS-MSQDs, including top-down and bottom-up methods. For top-down approaches, the lateral size of layered MS is reduced through physical or chemical methods, including a sequential combination of salt-assisted ball-milling and sonication-assisted solvent exfoliation method [27], an electrochemical approach [28], ultrafast laser ablation [29], a combination of a grinding/hydrothermal process and sonication [30], liquid exfoliation [31] and a sodium-ion intercalation-assisted approach [32] etc. In these methods, the in-plane chemical bonds of bulk MS are broken by external forces or chemical cutting processes and then the weak interlayer van der Waals interactions are broken by liquid exfoliation. Therefore, these approaches require time-consuming, rigorous conditions, tedious post-treatment and produce a low yield of the QDs. For bottom-up approaches, the WS-MSQDs are synthesized by hydrothermal and solvothermal method [33,34,35] using different molybdenum and sulfur sources. However these methods also suffer from cumbersome post-treatment. The above drawbacks impede their practical application. In our previous work, we developed an efficient bottom-up strategy for high-yield production of WS-MSQDs by a sintering/etching/exfoliation approach and the yield of WS-MSQDs is over 30% [36]. However, the water solubility of obtained MSQDs is poor. This disadvantage prejudices their biological applications.

In this paper, we report a simple and efficient method for large-scale production of WS-MSQDs by a bottom up strategy. The obtained WS-MSQDs exhibit sufficient water solubility and remarkable fluorescence properties, which have been utilized as a probe for detection of Fe^3+^ ions with high selectivity and specificity. Furthermore, the WS-MSQDs are used for the fluorescence imaging of Fe^3+^ in living cells successfully.

## 2. Experimental Section

### 2.1. Materials and Apparatus

All reagents or solvents were purchased from commercial providers and used without further purification. Transmission electron microscope (TEM) images were obtained from FEI Tecnai F30 microscope (FEI Tecnai F30, Hillsboro, OR, USA). Atomic force microscope (AFM) was performed on an Asylum Research MFP-3D instrument (Asylum Research, MFP-3D, Santa Barbara, CA, USA). The crystal structure properties of samples were characterized by an X-ray diffraction instrument (XRD, Phillips X’pert Pro, Almelo, the Netherlands). Raman spectra of the samples were performed on a micro-Raman spectroscope (JY-HR800, longjumeau, France). The X-ray photoelectron spectroscopy (XPS) spectra were measured spectrometer (ESCALAB210, VG, UK). The UV-vis spectra were recorded with an Agilent Cary 5000 spectrophotometer (Agilent Technologies, Palo Alto, CA, USA). The luminescence spectra were recorded using a Hitachi F-7000 spectrophotometer (Hitachi, Tokyo, Japan). All pH measurements were made with a pH-10C digital pH meter.

### 2.2. Synthesis of High Water-Solubility Molybdenum Disulfide Quantum Dots (WS-MSQDs)

The WS-MSQDs were synthesised through the methods describing in our previous paper with minor modification [36]. In order to improve the water solubility of the obtained MSQDs, the resulting powder after an etching process needed a solvothermal treatment. Briefly, 50 mg of the resulting powder was dispersed into 200 mL N, N-dimethylformamide (DMF). After sonicating in an ice bath for 3 h, the dispersion was kept stirring for 4 h at 140 °C. Afterwards, the stabilized deep yellow suspension containing amount of WS-MSQDs was obtained by centrifuging at 4000 rpm for 15 min. Finally, the WS-MSQDs powder was obtained by vacuum rotary evaporation and evaporated under vacuum at 80 °C. The yield of water soluble WS-MSQDs was about 30 wt %. Then WS-MSQDs powder was dispersed in deionized water for further applications. Redispersion of WS-MSQDs in water at 13.8 mg/mL showed high stability when standing still for one week. Hence, the WS-MSQDs exhibited sufficient water solubility and the maximum concentration of WS-MSQDs in water was 13.8 mg/mL.

### 2.3. Photoluminescence Measurement

All the fluorescence measurements were performed in HEPES (10 mM, pH 7.2) buffer solution at room temperature. Stock solution of metal ions including Fe^3+^, Ag^+^, Al^3+^, Ba^2+^, Ca^2+^, Cd^2+^, Co^2+^, Cr^3+^, Ga^3+^, Hg^2+^, K^+^, Li^+^, Mg^2+^, Mn^2+^, Na^+^, Ni^2+^, Pb^2+^ and Zn^2+^ in acetonitrile were prepared with an concentration of 10^−1^ M using their perchlorates, and stock solution of WS-MSQDs (100 μg/mL) in deionized water was prepared. In a typical assay, 200 μL WS-MSQDs stock solution was diluted in 1800 μL HEPES buffer solution at a final concentration of 10 μg/mL. For a cation competitiveness study, 10 μL of these metal ions were added into the above solution at a final concentration of 500 μM.

### 2.4. Cell Culture

BHK cells were seeded to the 12-well plates and cultured in culture media (Dulbecco’s Modified Eagle Medium) subjoined with 10% FBS (fetal bovine serum) at 37 °C in a humidified incubator containing 5% CO_2_. After 24 h, the cells incubated with 20 μg/mL WS-MSQDs for 1 h and then they were incubated with (100 μM) Fe(ClO_4_)_3_ for another 1 h. For the control experiment, BHK cells were incubated only with 20 μg/mL WS-MSQDs for 1 h under the same conditions. Before imaging measurement, the cells were rinsed three times with phosphate buffered saline.

## 3. Results and Discussion

### 3.1. Characterizations of MSQDs

MoCl_5_ (0.5 g) and thiourea (1 g) were used as molybdenum and sulfur source to synthesize WS-MSQDs via a sintering/etching/exfoliation process describing in our previous paper [36]. During the synthesis process, the amount of nano-SiO_2_ particles not only play the role of template, but also impede the growth of the MS crystal nucleus, which contribute to form MS nanoplates composed of large amounts of stripe-like MS grains with dimensions of several tens of nanometers. Hence, a lot of the obtained MS nanoplates can be easily transformed into quantum dots via a sonication-assisted solvent exfoliation method, which guarantees the improvement of the yield of MSQDs. However, the obtained MSQDs exhibit poor water solubility. To improve the water solubility of MSQDs, the obtained MS nanoplates were subjected to a solvothermal treatment and this process helped to cut MS nanosheets into quantum dots simultaneously. Then the supernatant containing amounts of WS-MSQDs was obtained by liquid exfoliating of MS nanoplates and centrifugation. Finally, the WS-MSQDs were obtained by vacuum rotary evaporation. By calculating the mass ratio of the eventual WS-MSQDs powder and the original MS nanoplates, the production yield of WS-MSQDs exceeded 30 wt %, which was higher than that reported previously [27].

**Figure 1:**
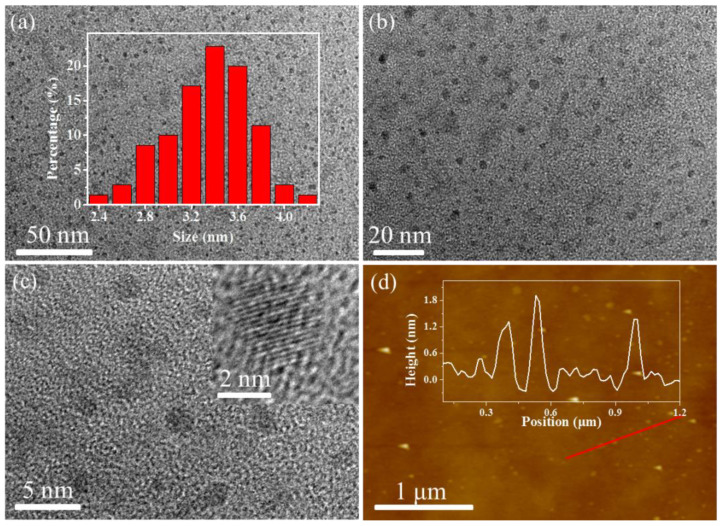
Morphology characterization of high water-solubility molybdenum disulfide quantum dots (WS-MSQDs). (**a**–**c**) Transmission electron microscope (TEM) images of WS-MSQDs. Inset of (**a**,**c**): the size distributions and high-resolution (HR) TEM pattern of the WS-MSQDs. (**d**) Atomic force microscope (AFM) image of the WS-MSQDs. Inset of (**d**): height profiles along the red lines in (**d**).

**Figure 2:**
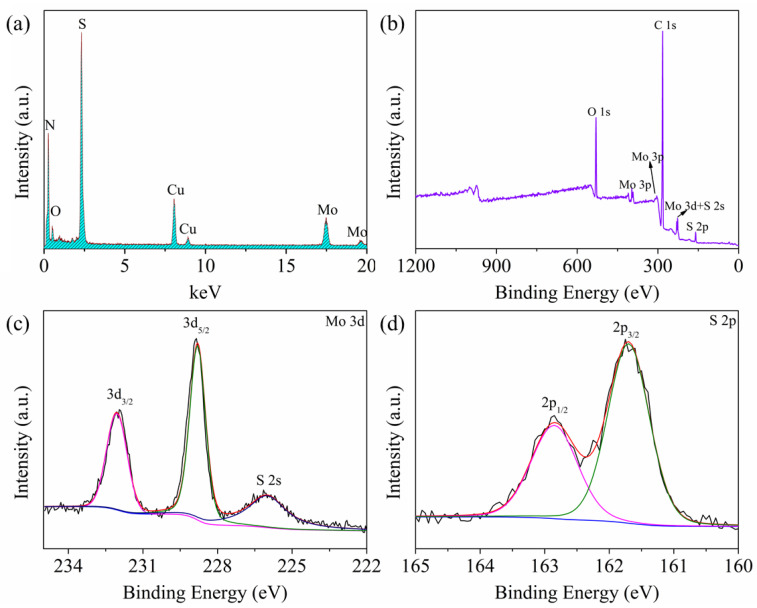
(**a**) Energy-dispersive X-ray spectrometry (EDX) spectrum of the WS-MSQDs. (**b**) X-ray photoelectron spectroscopy (XPS) image of the WS-MSQDs. The high-resolution XPS spectra of (**c**) Mo 3d and (**d**) S 2p.

The WS-MSQDs were characterized by transmission electron microscopy (TEM). TEM images (Figure 1a,b) show that the WS-MSQDs are uniformly distributed without aggregation and the average size is 3.4 nm. Furthermore, high-resolution TEM (HRTEM) images (Figure 1c) indicate the highly crystalline structure of the WS-MSQDs. The morphology and thickness of WS-MSQDs were investigated by an atomic force microscope (AFM), which confirmed the height of the WS-MSQDs varied from 0.6 to 1.8 nm. The energy-dispersive X-ray spectrometry (EDX) spectra, as shown in Figure 2a, indicate that only the elements of Mo and S are present in WS-MSQDs. The element Cu comes from the copper grid for TEM measurement. Furthermore, the chemical state and surface composition of WS-MSQDs were investigated by X-ray photoelectron spectroscopy (XPS) measurements (Figure 2b). As shown in Figure 2b, no peak for Si was detected, indicating that nano-SiO_2_ in the product were removed completely. Figure 2c,d depict the high-resolution XPS of the WS-MSQDs in the Mo 3d and S 2p. As shown in Figure 2c, the two main peaks at 232.1 and 228.8 eV corresponded to Mo^4+^ 3d_3/2_ and Mo^4+^ 3d_5/2_, respectively, and the peak at 225.9 eV is S 2s. As shown in Figure 2d, two peaks at 161.7 and 162.8 eV corresponded to S 2p_3/2_ and S 2p_1/2_, respectively [37]. These results indicated that the obtained WS-MSQDs are trigonal prismatic (2H) phase.

**Figure 3:**
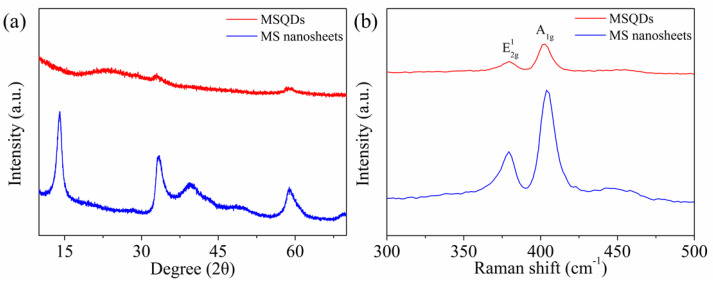
(**a**) X-ray diffraction (XRD) patterns and (**b**) Raman spectra of the MS nanosheets and WS-MSQDS.

The phase identity of the WS-MSQDs was confirmed by powder XRD and RAMAN spectroscopy measurements. Figure 3a depicts the XRD data for the WS-MSQDs and MS nanosheets is used as a reference. It shows that only two weak diffraction peaks are detected at 32.82° and 58.42° for WS-MSQDs, and most of the other peaks have disappeared. This demonstrates the formation of MONO- or FEW-LAYERED WS-MSQDs. Figure 3b shows that the Raman spectra for MS nanosheets presents two distinct peaks at 379.5 cm^−1^ and 403.4 cm^−1^ for the $E_{2g}^{1}$ and $A_{g}^{1}$ vibrational modes, respectively [38]. The $E_{2g}^{1}$ mode of the monolayer WS-MSQDs is red-shifted compared to that of MS nanosheets, which is identical with previous report [39].

### 3.2. Optical Properties of MSQDs

**Figure 4:**
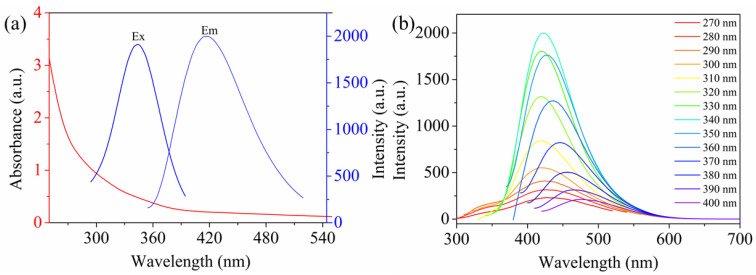
(**a**) Ultraviolet–visible (UV-vis) absorption and fluorescence emission spectrum of WS-MSQDs. (**b**) Fluorescence emission spectra of WS-MSQDs at different excitation wavelength.

The optical properties of the WS-MSQDs were explored in HEPES (10 mM, pH 7.2) buffer solution. The ultraviolet–visible (UV-vis) absorption spectra show an absorption peak at 295 nm, which corresponds to the excitonic feature of WS-MSQDs, presented in Figure 4a. The WS-MSQDs emit fluorescence at 425 nm upon the excitation of 340 nm with a quantum yield of 5.9%. Figure 4b shows the fluorescence emission spectra of the WS-MSQDs excited by light with different wavelengths. From it, we can see the fluorescence emission of WS-MSQDs exhibited an excitation-dependent behaviour with the excitation wavelength changing from 270 to 400 nm. This photoluminescence behavior is analogous to the previous report [39], which is generated from the high homogeneity and good water solubility of the WS-MSQDs. According to Figure 1a in the manuscript, the particle size of WS-MSQDs is ranging from 2.4 nm to 4.2 nm. It has been demonstrated that the luminescence properties of MSQDs depend on particle size [40], which means that WS-MSQDs with different particle sizes exhibit different emission wavelengths. Hence, the excitation-dependent behavior of the WS-MSQDs may be attributed to the polydispersity of WS-MSQDs. Furthermore, this behaviour may also be attributed to the hot fluorescence from the K point of the Brillouin zone [41,42].

### 3.3. Effects of pH

**Figure 5:**
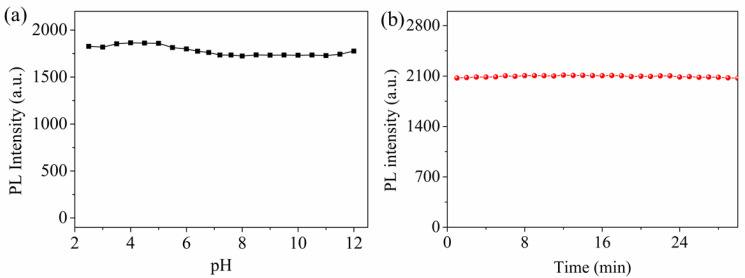
The photostability of WS-MSQDs (**a**) Fluorescence stability studies of WS-MSQDs in different pH solutions. (**b**) Fluorescence intensity of WS-MSQDs under excitation at 340 nm for 30 min.

The stability of the fluorescence for WS-MSQDs under different conditions was also explored. As shown in Figure 5a, the fluorescence intensity of WS-MSQDs is found to be independent of pH over a wide range (2.5–11.0), which indicates that the influence of pH on the fluorescence of WS-MSQDs is negligible. Furthermore, the fluorescence intensity of WS-MSQDs has no significant decline under continuous irradiating at 340 nm for 30 min (Figure 5b), which indicates that WS-MSQDs possess excellent anti-photo bleaching capability and photobleaching does not occur. These results indicate that the WS-MSQDs exhibit excellent photostability. The reason for the excellent photostability of WS-MSQDs may be that WS-MSQDs are not easily affected by acidic, alkaline or irradiation conditions, which means that the chemical stability of WS-MSQDs in these conditions guarantees the excellent photostability.

### 3.4. Detection of Fe^3+^ Ions and Selectivity Measurements

**Figure 6:**
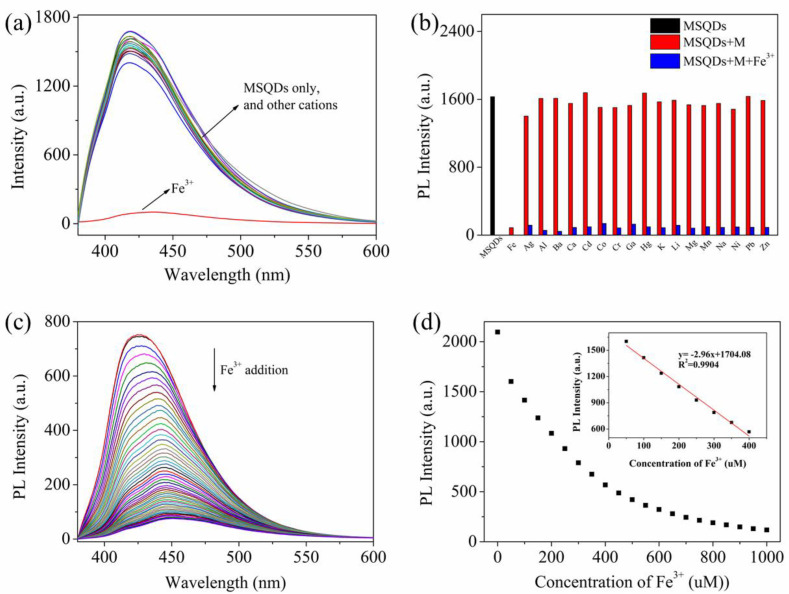
(**a**) Fluorescence spectra of WS-MSQDs upon addition of various metal ions (Fe^3+^, Ag^+^, Al^3+^, Ba^2+^, Ca^2+^, Cd^2+^, Co^2+^, Cr^3+^, Ga^3+^, Hg^2+^, K^+^, Li^+^, Mg^2+^, Mn^2+^, Na^+^, Ni^2+^, Pb^2+^ and Zn^2+^). (**b**) Relative fluorescence intensities of WS-MSQDs at 425 nm. (black bars: WS-MSQDs; red bars: WS-MSQDs with other metals; blue bars: WS-MSQDs with other metals ions and Fe^3+^ ions). (**c**) Fluorescence spectra of WS-MSQDs in the presence of different concentration of Fe^3+^ ions. (**d**) Fluorescence intensity of WS-MSQDs versus increasing concentrations of Fe^3+^ ions. Inset of (**d**): The linear changes of fluorescence intensity of WS-MSQDs at 425 nm upon titration with Fe^3+^ ions. All spectra were acquired in HEPES (10 mM, pH 7.2) buffer solution at room temperature. [WS-MSQDs] = 10 μg/mL, *λ*_ex_ = 340 nm, Slit: 5.0 nm/5.0 nm.

To evaluate the recognition capability of WS-MSQDs towards Fe^3+^ ions over other metal ions (such as Ag^+^, Al^3+^, Ba^2+^, Ca^2+^, Cd^2+^, Co^2+^, Cr^3+^, Ga^3+^, Hg^2+^, K^+^, Li^+^, Mg^2+^, Mn^2+^, Na^+^, Ni^2+^, Pb^2+^ and Zn^2+^), a selectivity experiment was also carried out. As shown in Figure 6a, only the addition of Fe^3+^ results in significant quenching effect on the fluorescence of WS-MSQDs, whereas no obvious changes are observed upon the addition of other metal ions under the same conditions, which indicates the high selectivity of WS-MSQDs for Fe^3+^ in aqueous solutions and potential as an effective fluorescence probe for Fe^3+^ detection. Furthermore, the cation-competitive experiments were conducted in the presence Fe^3+^ ions mixed with different metal ions, as shown in Figure 6b. As a result, the fluorescence intensity has changed little under the condition of these ions coexistence, suggesting the competing ions have tiny influence on the fluorescence intensity of WS-MSQDs.

Then, the sensing performance of WS-MSQDs towards Fe^3+^ was investigated systematically in HEPES (10 mM, pH 7.2) buffer solution. The fluorescence titration spectra of Fe^3+^ to WS-MSQDs are displayed in Figure 6c. Upon addition of different concentrations of Fe^3+^ ions, the fluorescence intensity of the WS-MSQDs at 425 nm decreases gradually linearly. From the linear equation (Figure 6d), the detection limit (LOD) for Fe^3+^ ions was measured to be 2.03 μM (3σ per slope) (R^2^ = 0.9904), which met the limits of Fe^3+^ in drinking water (5.357 μM) set by the U.S. Environmental Protection Agency [43]. Therefore, WS-MSQDs could serve as a fluorescence turn-off probe for quantitative detection of Fe^3+^ ions.

### 3.5. Fluorescence Imaging

**Figure 7:**
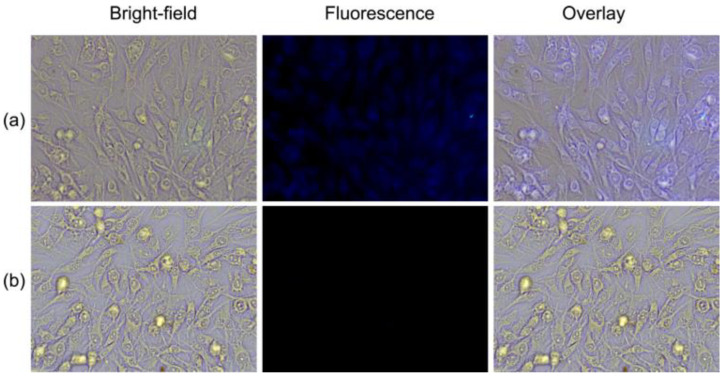
Bright-field and fluorescence images of BHK cells. (**a**) BHK cells were incubated with WS-MSQDs (20 μg/mL) for 1 h. (**b**) BHK cells were incubated with WS-MSQDs (20 μg/mL) for 1 h and then further incubated with Fe^3+^ (100 μM) for 1 h.

In addition, considering the positive results in vitro, the fluorescence imaging of WS-MSQDs for Fe^3+^ in living cells was studied. As shown in Figure 7a, an intense intracellular blue fluorescence could be seen when BHK cells were incubated with WS-MSQDs (20 μg/mL) for 1 h at 37 °C, implying that WS-MSQDs possessed good cell membrane permeability. However, the cells treated with WS-MSQDs (20 μg/mL) were further incubated with Fe^3+^ (100 μM) for another 1 h (Figure 7b), and an obvious fluorescence decrease was observed, which was in agreement with the Fe^3+^ induced fluorescence response. Taken together, WS-MSQDs was biocompatible and suitable for imaging of Fe^3+^ in living cells.

## 4. Conclusions

In summary, mass production of WS-MSQDs was achieved via a sequential combination of a sintering/etching/exfoliation method and a solvothermal route. With such a strategy, uniform WS-MSQDs were produced with a high yield of more than 30 wt %, indicating the significant competitiveness of the synthetic method that we proposed toward mass production of the WS-MSQDs. The obtained WS-MSQDs with an average size of 3.4 nm display sufficient water solubility and remarkable fluorescence properties. Furthermore, the WS-MSQDs were utilized as a probe for the selective and sensitive detection of Fe^3+^ ions. The WS-MSQDs exhibit high fluorescence stability under a wide range of pH and continuous irradiation. Finally, the WS-MSQDs were used for the fluorescence imaging of Fe^3+^ in living cells, which exhibited practical potential for various bio-applications.
